# Prevalence of Cytomegalovirus in Semen of Male Partners of Infertile Couples and the Virus Impact on Sperm Parameters

**Published:** 2020

**Authors:** Bahia Namavar Jahromi, Ramin Yaghobi, Najmeh Matlub, Arezou Fazelzadeh, Abolfazl Ramzi, Zahra Anvar, Najaf Zare, Leila Salarian, Jafar Fallahi

**Affiliations:** 1- Infertility Research Center, Shiraz University of Medical Sciences, Shiraz, Iran; 2- Department of Obstetrics and Gynecology, School of Medicine, Shiraz University of Medical Sciences, Shiraz, Iran; 3- Maternal-Fetal Medicine Research Center, Shiraz University of Medical Sciences, Shiraz, Iran; 4- Shiraz Transplant Research Center, Shiraz University of Medical Sciences, Shiraz, Iran; 5- IVF Section, Ghadir Mother and Child Hospital of Shiraz, Shiraz, Iran; 6- Department of Biostatistics, Shiraz University of Medical Sciences, Shiraz, Iran; 7- Department of Pediatrics, Shiraz University of Medical Sciences, Shiraz, Iran; 8- Molecular Medicine Department, School of Advanced Medical Sciences and Technologies, Shiraz University of Medical Sciences, Shiraz, Iran

**Keywords:** Cytomegalovirus, Male infertility, Polymerase chain reaction, Semen analysis

## Abstract

**Background:**

Genital tract infection is one of the causes of male infertility. Several studies have shown a role for human cytomegalovirus (CMV) in this context. In the present study, the prevalence of CMV in a population of male partners of infertile couples was estimated and the impact of CMV on sperm parameters was determined.

**Methods:**

In this cross sectional study, CMV DNA and virus copy number were examined in the semen of 150 participants including 80 with normal semen analysis (SA) and 70 with abnormal SA, by quantitative Real-Time PCR. Sperm parameters were compared between CMV positive and negative groups. Comparisons with p- values under 0.05 were considered significant. Logistic regression was performed to control the effect of some variables with p<0.25 on sperm parameters.

**Results:**

CMV DNA was detected in the semen of 28 (18.6%) individuals. 21 men (30%) with abnormal SA and 7 (8.8%) with normal SA were positive for CMV DNA (p=0.001). The mean virus copy number was 883.1±4662.01 for the men with abnormal SA and 2525.7±12680.9 for those with normal SA (p=0.001). Sperm count was (32.1±23.5) ×10^6^ in CMV positive and (44.2±24.1) ×10^6^ in CMV negative groups (p=0.022). Normal sperm morphology was 2.73±2.83% and 5.99±5.44% in CMV positive and negative groups, respectively (p<0.001). After controlling some variables, the sperm morphology remains the only statistically significant sperm parameter that was reduced by CMV.

**Conclusion:**

The higher CMV prevalence in the semen of males with abnormal SA compared to normal SA and significant reduction of sperm morphology in the presence of CMV, are in favor of the negative impact of CMV on male fertility.

## Introduction

Cytomegalovirus (CMV), a double–stranded DNA virus belongs to a family called herpesviridae or human herpesviruses (HHVs) (1, 2). CMV is spread by direct contact withinfectious body fluids such as nasal secretions, saliva, tears, urine, genital secretion or breast milk (3). After contamination, the virus initially replicates in the epithelial cells at the site of entry, followed by hematogenous spread to numerous organs and cell types. CMV persists life–long in infected individuals, with the genome detected in stem cells, myeloid precursor cells and monocytes (4).

CMV contamination rate is higher among children and young adults. Moreover, among males, this rate increases by the age. Other risk factors in adolescent males include the ethnicity (African Americans more than other races), some risky personal behaviors and living in either group living or crowded situations (5).

Infertility is defined as the inability to conceive a child after one year of unprotected intercourse. Approximately 15% of couples of reproductive age worldwide suffer from infertility and male factors account for half of them (6–8). In Iran, the prevalence of infertility is estimated to be 10.9% and male factor infertility accounts for 34% of all cases (9).

Genital tract infections in human are proven to be one of causes of infertility. Sexually transmitted diseases (STDs) in men cause genital injury, infections of semen, prostatitis, urethritis, epididymitis and orchitis (10).

Viral infections of male genital tract have been investigated for years as possible causes of male infertility (11). There are several mechanisms by which viruses might influence male infertility including direct effect on spermatogenesis resulting in sperm dysfunction, inflammatory changes in the composition of genital secretions and induction of immune response by production of anti– sperm antibodies (12).

In the literature, there are some discrepancies among studies showing the relationship between HHV infections (Including CMV, herpes simplex virus (HSV) and Epstein-Barr virus (EBV)) and sperm parameters or male infertility (13–16), with some studies confirming these relationship and some studies rejecting the same. For instance, in one of the studies with the impact of HHV infections on sperm parameters, it has been shown that the DNA of STD pathogens in semen was associated with reduced sperm count and motility (15).

In this project we aimed to determine the prevalence of CMV in the semen of male partners of infertile couple attending Ghadir Mother and Child hospital. The PCR method was used because immunohistochemistry (IHC) and PCR are often considered the most sensitive tests for detecting CMV. Furthermore, the association between presence of CMV in semen and sperm parameters was assessed.

## Methods

### Selection of participants:

The study was performed on 150 males, who attended the infertility center of Ghadir Mother and Child hospital, affiliated to Shiraz University of Medical Sciences. The sample recruitment was done between February 2015 and March 2016. According to WHO 2010, a semen sample was considered normal when fitting the following criteria: count ≥15 *million/ml*, morphology ≥4%, and motility ≥32% (17). If at least one of the above criteria was not present, the sample was considered as abnormal SA. Accordingly, 80 individuals had normal SA and 70 individuals had abnormal SA. Individuals with the age under 20 or over 55, concurrent malignancy, receiving medication for infertility were excluded from this study. The study was explained to all patients and informed consent was taken from all participants and the local ethics and scientific committee of Shiraz University of Medical Sciences approved the study (Ethics code: IR.SUMS. REC.1393.6917).

### Semen analysis and DNA extraction:

Semen sample was collected by masturbation in sterilized containers. Samples were immediately kept in an incubator and after liquefaction underwent microscopic examination. Around 200 *μl* of each sample was kept at −80°*C* until the time of DNA extraction. DNA was extracted using Invisorb Spin Virus DNA Mini Kit (Stratec, Germany).

### Quantitative real-time PCR:

The presence and level of genomic CMV DNA was evaluated in studied samples using genesig real-time PCR kit (Primer Design Ltd TM, Advanced kit, United Kingdom). The reaction mix for PCR was performed in 20 *μl* total volume and the program used for this reaction was 1 cycle 95*°C* for 10 *min*, followed by 50 cycles of 95*°C* for 5 *s* and 60*°C* for 60 *s* using Step One Plus Real-Time Thermocycler (Applied Biosystems, USA). The quality of quantitative real-time PCR was checked using pre-qualified and confirmed CMV negative and positive controls.

### Statistical Analysis:

The sample size was calculated to be 71 cases in each group, by considering the significance level of 0.05, power of 0.80, in addition to 8% and 25% positive CMV rate in infertile men with normal and abnormal SA, respectively according to the previous studies and our pilot study.

SPSS 17 for windows was used for analysis. The comparison between groups for prevalence of CMV infection was done by Chi-Square test and the effect of presence of CMV on sperm parameters was done by two-tailed t-test. The p- values under 0.05 were considered to be significant. To control the effect of some confounding variables with p<0.25, logistic regression was used.

## Results

150 male partners of infertile couples were enrolled in this study. Demographic data of participants according to presence or absence of CMV are compared in table 1.

**Table 1.** Comparison of demographic data between CMV positive and CMV negative groups

	**CMV positive (28)**	**CMV negative (122)**	**p-value**
**Age (years)**	38.9±7.29	37.6±6.94	0.362
**BMI (*kg/m*^2^)**	24.7±3.73	24.7±2.97	0.990
**Family history of infertility**	7 (24 %)	22 (76%)	0.400
**Previous trauma to genitalia**	5 (25%)	15 (75%)	0.536
**Previous mumps**	7 (11.5%)	54 (88.5%)	0.061
**Varicocele surgery**	6 (28.6%)	15 (71.4%)	0.230
**Medications**	2 (18.2%)	9 (81.8%)	0.966
**Smoking**	8 (17.4%)	38 (82.6%)	0.968
**Abdominal and genital surgery**	7 (24.2%)	22 (75.8%)	0.409

CMV: Cytomegalovirus. BMI: Body Mass Index. Data are presented as N (%) or mean±SD

Table 2 shows the presence of CMV and virus copy number compared between normal SA and abnormal SA groups.

**Table 2.** Comparison of virus presence and copy number between normal and abnormal semen groups

	**Normal SA (80)**	**Abnormal SA (70)**	**p-value**
**CMV positive**	7 (8.8%)	21 (30%)	0.001
**Mean virus copy number in semen**	883.1±4662.01	2525.7±12680.9	0.001

CMV: Cytomegalovirus. SA: Semen Analysis. Data are presented as N (%) or mean±SD

Sperm parameters in association with the presence or absence of virus were investigated among all participants. Data are shown in table 3.

**Table 3.** Comparison of sperm parameters between CMV positive and CMV negative groups

	**CMV positive (28)**	**CMV negative (122)**	**p-value**
**Count (×106/*ml*)**	32.1±23.5	44.2±24.1	0.022
**Morphology (%)**	2.73±2.83	5.99±5.44	≤0.001
**Motility (%)**	38.1±14.4	42.8±14.2	0.068

CMV: Cytomegalovirus. Data are presented as mean±SD

To control the effect of two variables with p- value under 0.25 shown in table 1, logistic regression was performed. Table 4 shows the results.

**Table 4.** Results of logistic regression on sperm parameters and previous mumps or varicocele surgery

	**B**	**SE**	**p-value**	**Odds ratio (95% CI)**
**Morphology**	−0.220	0.111	0.047	0.803 (0.646–0.998)
**Motility**	0.008	0.020	0.672	1.008 (0.970–1.048)
**Count**	−0.015	0.012	0.208	0.985 (0.962–1.009)
**Varicocele surgery**	0.471	0.583	0.419	1.601 (0.511–5.021)
**Previous mumps**	−1.090	0.532	0.040	0.336 (0.118–0.954)
**Constant**	−0.093	0.772	0.904	0.911 (0.201–4.138)

CI: Confidence Interval

## Discussion

CMV seroprevalence rate in Iran is estimated to be up to 98% (18, 19). CMV prevalence in semen is largely variable worldwide (11, 15, 20, 21). In Iran, CMV prevalence in semen is reported to be 1.4% to 15% in infertile populations in different centers (1, 16, 22, 23). In our study performed in Ghadir Mother and Child hospital infertility center, the prevalence of CMV in semen was estimated to be 18.6%.

The impact of CMV on male fertility and/or sperm parameters is discussed in numerous studies and certain discrepancies exist. Some studies rejected any association between CMV presence in the semen and male infertility (1, 12, 14, 21, 24), whereas some others debated a positive correlation (11, 15, 20, 22).

Our results showed a correlation between CMV presence in semen and male infertility. In our studied population, CMV positive and negative individuals were compared for some demographic variables. None of the variables was significantly different among positive and negative groups.

It was found that the prevalence of CMV and the virus copy number in semen among males with abnormal SA were almost three folds more than those with normal SA, confirming the relationship between CMV and male factor infertility. Moreover, sperm count, morphology and motility were lower in CMV positive group compared to negative group. The reduction was statistically significant for sperm count and morphology. However, to control the most effective variables with p<0.25 (Previous mumps and varicocele surgery), logistic regression was performed. This regulation showed that sperm morphology was still significantly reduced by CMV. However, the sperm count, which initially showed statistical significant value, was probably masked by others variables (As shown in table 4). Interestingly, it was found that previous mumps is a significant confounder influencing sperm parameters. The finding is in agreement with previous data (25).

CMV is able to replicate in male germ cells and so is proposed to contribute to male infertility; also CMV is easily transmissible by the infected semen to the partner (11). Moreover, the vertical CMV transmission from mother to the fetus is a threatening factor for the fetus, which might occasionally lead to symptomatic congenital CMV infection (cCMV) (26). Congenital CMV (cCMV) infection is the most common congenital infection, occurring in 1 per 150 live births. Approximately 10% of neonates with cCMV have symptomatic manifestations at birth, such as intrauterine growth retardation, hepatomegaly and microcephaly which can lead to neurodevelopmental complications including mental retardation and sensorineural hearing loss (SNHL) (27). However, most infected newborns (85–90%) are asymptomatic. Among them, 10–15% will develop SNHL or other permanent sequelae when grow up. The current targeted standard screening program for cCMV includes direct detection of virus in a saliva or urine sample by PCR in the first 2–3 weeks of age. Saliva PCR testing showed high sensitivity (97–100%) and specificity (99.9%) as a cCMV screening method (28). However, saliva should be collected at least one hour after the baby is breastfed for elimination of the risk of false positive results. Also, urine PCR as a confirmatory procedure can also be used for eliminating the risk of CMV shedding in the breast milk of CMV seropositive mothers (29, 30). Antiviral treatment of selected newborns with valganciclovir for 6 months appears to effectively improve hearing and neurocognitive outcomes (31).

Although there are reports on the use of antiviral medications for the management of CMV viremia but currently no proven treatments or particular vaccine is available for CMV and no data address the efficacy of preventive strategies (32). Therefore, despite the proven negative effect of CMV infection on general health of population, unfortunately no definite management is available. So, it seems that discovery and implementation of CMV vaccine for the general population in the future may effectively prevent the adverse CMV effects.

The present study has some limitations, *e.g*. small sample size. Future studies with larger sample sizes and treatment of the CMV semen positive men with antiviral medications accompanied by comparison of the semen parameters before and after treatment are recommended. Also, simultaneous evaluation of CMV in blood or other germ cells and direct comparison between virus copy number and each sperm parameter are suggested.

## Conclusion

Our results support the negative effect for the presence of CMV DNA in semen on sperm parameters specially sperm morphology and suggest for CMV involvement in male infertility.
